# Water content and cholesterol dynamics in various chicken meat cuts influenced by thermal processing methods: A multi-city assessment

**DOI:** 10.5455/javar.2026.m1017

**Published:** 2026-03-10

**Authors:** Dian Sundari, Nunung Nurjanah, Efriwati Efriwati, Fitrah Ernawati, Fifi Retiaty, Suharmiati Suharmiati, Aya Yuriestia Arifin, Elisa Diana Julianti, Putri Reno Intan, Sukmayati Alegantina, Budi Setyawati, Salimar Salimar

**Affiliations:** 1 Center for Public Health and Nutrition Research, National Research and Innovation Agency, Cibinong Science Center, Bogor 16911, West Java, Indonesia; 2 Research Center for Pharmaceutical Ingredients and Traditional Medicine, National Research and Innovation Agency, Cibinong Science Center, Bogor 16911, West Java, Indonesia; 3 Center for Biomedical Research, Research Organization for Health, National Research and Innovation Agency, Cibinong Science Center, Bogor 16911, West Java, Indonesia

**Keywords:** Chicken meat, sampling city, various processing, cholesterol content

## Abstract

**Objectives:** This study aimed to evaluate the water content and cholesterol levels in fresh and processed chicken meat from four major cities in Indonesia using various cooking methods.

**Materials and Methods:** Four major Indonesian cities (Medan, Bandung, Surabaya, and Makassar) were the sites of a purposeful sampling of four different parts of chicken meat: whole, breast, thigh, and wing. Meat was analyzed fresh and processed (boiled, fried, steamed, baked, and grilled). Saponification and toluene extraction procedures were used, and the amount of water was determined by oven-drying at 100–105°C. To determine total cholesterol levels, gas chromatography with flame ionization detection (GC-FID) and gas chromatography-mass spectrometry (GC-MS) methods were used. The study employed a completely randomized design, ANOVA, and Duncan’s test, and SPSS version 26 software was used to perform statistical analysis at a 95% confidence level.

**Results:** Water content and cholesterol levels of chicken meat were significantly influenced by body parts and processing methods. Fresh chicken had the highest water content, while frying caused the greatest moisture loss. Processed chicken, especially fried and baked whole and wing meat, showed higher cholesterol levels than fresh samples. Processing methods had a greater effect than differences among cities, as confirmed by ANOVA.

**Conclusions:** The highest water content was observed in fresh chicken breasts. In fresh samples, the thigh had the highest cholesterol content, whereas the whole part had the lowest. Among the processing methods, frying resulted in the greatest reduction in water content and the highest increase in cholesterol levels. Boiling was the most effective method for minimizing cholesterol increases. Although some differences in cholesterol levels were observed between cities, the processing method had a more pronounced effect than the geographic origin.

## 1. Introduction

Chicken meat is healthy because it contains carbohydrates, proteins, fats, amino acids, vitamins, minerals, and other beneficial nutrients [1, 2, 3]. The protein content of chicken meat is high because it contains all the amino acids the body needs and is easily digested and absorbed [4]. Chicken is tasty, smells good, soft, and not too expensive. People in Indonesia are more aware of the importance of eating healthy foods rich in animal protein [5]. The most common source of high-quality protein is chicken meat [6]. In 2018, people consumed 5,579 kg of broiler chicken meat per person, and by 2021, that number had increased to 8.1 kg per person [7, 8].

Chicken meat comprises 16–22% protein, 1.5–13% fat, 65–80%water, 1% inorganic compounds, 0.5% carbohydrates, and 1.5% non-protein nitrogen (NPN) [9]. Meat, including poultry, contains cholesterol, which can increase its levels in the body [10]. Chicken meat is low in fat, but cholesterol levels depend on the part of the chicken consumed and how it is processed [11]. Excessive meat consumption can increase cholesterol levels in the bloodstream. Cholesterol can then be mixed with other substances to form plaques that block blood flow. This plaque can lead to stroke, carotid artery disease, and peripheral artery disease [12, 13, 14].

Food requires water for consumption. All food components, whether derived from animal or plant sources, have varying water content [15]. Determining water content is the most important and common test in food processing and testing [16, 17]. The amount of water in food ingredients is one of the most important aspects, as it can significantly impact the appearance, feel, and taste of food and determine its freshness and shelf life. A high water content in food makes it easier for bacteria, mold, and yeast to grow, which changes the ingredients [18]. Fresh (raw) chicken meat contains 66% water. According to previous studies, the water content of chicken meat ranges from 65.48% to 71.27% [19].

The nutritional value of food ingredients has a significant impact on health, particularly the fat and cholesterol contents. Meat products are a major source of saturated dietary fats. Consumption of meat products naturally increases total and low-density lipoprotein (LDL) cholesterol levels [20].

Cholesterol is a lipid (fat) derivative classified as a steroid or sterol and is associated with other fatty acids as esters. Cholesterol is an important component of the membranes that make up all cells and is a major component of neuronal and cerebral cells. Cholesterol is found in high amounts in glandular tissue and the liver, where it is synthesized and stored [21].

Cholesterol is usually produced in the body in appropriate amounts. However, it can increase owing to the consumption of animal fats, including chicken, eggs, duck, beef, goat, milk, freshwater fish, crabs, shrimp, shellfish, eels, and squid. The cholesterol content in chicken meat is 60 mg/100 gm, and the fat content is 25 gm/100 gm [22]. Diseases caused by high cholesterol levels include atherosclerosis (narrowing of blood vessels), coronary heart disease, stroke, and hypertension [23].

A diet high in saturated fat and cholesterol increases the risk of coronary heart disease and atherosclerosis [24]. Many studies have reported a correlation between high blood cholesterol levels and the risk of heart disease [25]. Total blood cholesterol levels should be < 200 mg/dl; if ≥ 200 mg/dl, the risk of heart disease increases [23]. Current guidelines for dietary cholesterol state that one should consume 200–300 mg of cholesterol per day [26].

Heating is a food processing method. Heating significantly affects the nutritional value of food, reducing its lipid content. The level of food damage varies with temperature and processing duration. The higher the temperature, the more intense the fat damage. One cause of fat damage is oxidation [19, 27]. In general, animal fat is considered unhealthy because of its high saturated fatty acid and cholesterol content. Poultry is preferred over other meats because it contains higher levels of polyunsaturated fatty acids (PUFAs). This study aimed to evaluate the water content and cholesterol levels in fresh and processed chicken meat from four major cities in Indonesia using various cooking methods.

## 2. Materials and Methods

### 2.1. Ethical approval

This study was approved by the Health Research Ethics Commission, Health Research and Development Agency, Ministry of Health of Indonesia, under ethics approval number LB.02.01/KE.162/2020.

### 2.2. Sample collection method

Fresh raw chicken meat was used as the study material. Sampling was performed according to a previously described protocol [28]. Fresh raw chicken meat was collected from the three largest traditional markets in four provincial capitals, which are major cities in Indonesia: Makassar (South Sulawesi), Medan (North Sumatra), Bandung (West Java), and Surabaya (East Java). The selection of these four cities was representative of Indonesia’s major cities. The traditional market, commonly known as the primary market, offers a wide range of bulk food ingredients.

Chicken meat samples, consisting of four types of chicken parts, were purchased at 3 kg each from traders who provided cuts weighing less than 50 kg. The four parts of chicken sampled in this study were as follows: 1. Whole chicken meat samples (without/excluding the head, neck, feet, and innards); 2. Chicken breast meat samples, 3. Chicken thigh meat samples, and 4. Chicken wing meat samples. Sampling was conducted purposively from 2 (two) traders in each market. Each chicken meat sample purchased from the first trader in each market was composited, as were the samples purchased from the 2nd trader in each market, so that the fresh/raw chicken meat samples in fresh condition were used as samples for repetition 1 (sample from trader 1) and samples for repetition 2 (sample from trader 2).

The fresh chicken meat samples that were purchased were not only taken as unprocessed samples but also as chicken meat samples that were processed by cooking with five basic processing methods into prepared food that is commonly done by Indonesian people, namely, frying, boiling, steaming, grilling, and roasting, resulting in six treatments. Each treatment was repeated 2 times, and each repetition was analyzed in duplicate. The collection, processing, and preparation of chicken meat samples were performed at the Food Chemistry Laboratory, Center for Biomedical and Basic Health Technology. This study used a completely randomized design. Statistical analyses were performed using SPSS version 26, followed by Duncan’s test at a 95% confidence level [29].

The chicken meat samples were processed as follows: 1) Unprocessed: Chicken meat samples that did not receive any treatment or were fresh. 2) Boiling: The samples were processed using boiling water in a closed pan. The samples were submerged in boiling water until cooked. 3) Fried: Processed samples using heated oil. The food ingredients were immersed in hot oil until they were cooked. 4) Steam: Samples were processed using boiling water vapor until cooking. If water for steaming is lacking, but the ingredients are not yet cooked, hot water can be added to the mixture. 5) Bake: Samples were processed using an oven or Teflon pan without adding water or oil, and 6) Grilling: Samples were processed by heating over a direct fire. The samples were grilled until cooked [30].

### 2.3. The determination of the water content of chicken meat

Analysis of water and cholesterol content was carried out using PT. Qualis Indonesia Laboratory, which has been accredited by the National Accreditation Committee with the number LP-708-IDN, and the International Laboratory Accreditation Cooperation Mutual Recognition Arrangement (ILAC MRA). Water content analysis used the AOAC 2006 method, and cholesterol analysis used the AOAC 994.10 method using GC-FID/GC-MS.

### 2.4. Determination of water content

The water content of the chicken meat was determined by oven drying. Homogenized chicken meat (2 gm) was accurately weighed using an analytical balance (± 0.1 mg) and placed in a porcelain crucible previously dried to a constant weight (W_c_). The combined weight of the crucible and sample before drying was recorded as W_c+s_.

The sample was dried in an oven at 100–105°C for 2 h, cooled in a desiccator, and weighed. Drying, cooling, and weighing were repeated at 1-h intervals until a constant final weight (W_f_) was achieved, defined as a weight change of less than 5 mg.

The water content was calculated using the following equation:


1 \[
\%{\rm M} = \frac{{{{\rm W}_{{\rm c} + {\rm s}}} - {{\rm W}_{\rm f}}}}{{{{\rm W}_{{\rm c} + {\rm s}}} - {{\rm W}_{\rm c}}}} \times 100{\rm \%}
\]

W_c_ = Weight of the empty crucible (gm)

W_c+s_ = Weight of the crucible and sample before drying (gm)

W_f_ = Weight of the crucible and sample after drying (gm)

### 2.5. Determination of total cholesterol content

Total cholesterol in chicken meat was determined using a saponification–extraction method followed by GC-FID/GC-MS analysis. Approximately 5 gm of the homogenized sample was saponified with 95% ethanol and 50% (w/v) KOH under ultrasonic agitation at 70°C for 10 min. After cooling to room temperature, the unsaponifiable fraction containing cholesterol was extracted with toluene under agitation and separated by centrifugation (4100 rpm, 5 min, 4°C).

The toluene layer was sequentially washed with 1 N KOH and distilled water until a clear phase was obtained. An aliquot of the extract was evaporated to dryness using a rotary evaporator at 40 ± 3°C, reconstituted in dimethylformamide (DMF), and adjusted to the working concentration.

Cholesterol was derivatized with trimethylchlorosilane (TCMS). Derivatized samples and cholesterol standards (5–100 mg/l) were spiked with 5-α-cholestane as an internal standard and analyzed using GC-FID/GC-MS within 24 h.

Cholesterol content was calculated from the GC analysis results and expressed as mg per 100 gm of sample.

### 2.6. Calculation

The mass of the sample per milliliter of the derivative solution was calculated using the following equation:


2 \[
{\rm Grams\ of\ sample\ per\ ml\ of\ derivative} =
\frac{{{\raise0.7ex\hbox{W1} \!\mathord{\left/
{\vphantom {{W1} {V1}}}\right.\kern-1pt}
\!\lower0.8ex\hbox{V1}}}}{{{\raise0.7ex\hbox{V2} \!\mathord{\left/
{\vphantom {{V2} {V3}}}\right.\kern-1pt}
\!\lower0.8ex\hbox{V3}}}}
\]

W1 = Weight of the sample (gm)

V1 = Volume of toluene used in the extraction (20 ml)

V2 = Volume of extract aliquot taken for drying (5 ml)

V3 = Volume of DMF used to dissolve the residue (3 ml)

Total cholesterol content was calculated as:


3 \[
{\rm Cholesterol}({\rm mg})/100{\rm gmsample} = \frac{{{\rm Cholesterolconcentration}}}{{{\rm Derivatizedsampleweight}({\rm gm}/{\rm ml})}} = \times 100
\]

## 3. Results

The results of the analysis of the water and cholesterol content of chicken meat, as many as 380 samples from four major cities in Indonesia, based on the body parts analyzed, namely whole chicken meat (without head, neck, feet, and innards), chicken breast meat, chicken thigh meat, and chicken wing meat with six processing treatments, are shown in the tables below.

### 3.1. The water content analysis

The analysis of water content in chicken meat (Table 1) showed the water content for various chicken parts (whole, breast, thigh, and wing) and processing methods (fresh, boiled, fried, steamed, grilled, and baked) in Medan, Bandung, Surabaya, and Makassar. In general, the highest water content was found in fresh chicken, whereas the lowest was in fried chicken. The average water content decreased significantly after processing, particularly with the frying method. Differences in water content were also observed between cities, with Medan generally having a higher water content than other cities. ANOVA results showed significant differences (indicated by different superscripts) between treatments and locations.

**Table 1.** The average water content of chicken meat from the four cities with different processing.

Part of Chicken Meat	Processing types	Moisture Content (%)				
		Medan	Bandung	Surabaya	Makasar	Average**
Whole	Fresh	73.95 ± 0.49	69.55 ± 0.64	67.80 ± 0.71	66.75 ± 1.48	69.51 ± 3.02^a^
	Boiled	63.14 ± 1.78	64.55 ± 1.34	64.20 ± 0.14	63.30 ± 1.13	63.80 ± 1.14^b^
	Fried	51.80 ± 0.00	54.20 ± 0.85	53.30 ± 0.42	52.60 ± 1.84	52.98 ± 1.23^e^
	Steamed	63.65 ± 0.40	63.35 ± 0.21	62.55 ± 0.21	62.05 ± 0.07	62.90 ± 0.70^b^
	Baked	57.75 ± 2.33	57.75 ± 2.33	59.70 ± 1.70	56.60 ± 4.81	57.95 ± 2.59^d^
	Grilled	59.60 ± 0.57	61.50 ± 0.71	60.55 ± 1.06	57.85 ± 0.49	59.88 ± 1.55^c^
	Average*	61.65 ± 7.12^a^	61.82 ± 5.20^a^	61.35 ± 4.70^a^	59.86 ± 5.16^b^	61.17 ± 5.50
Breast	Fresh	73.78 ± 0.34	70.90 ± 1.70	67.93 ± 0.04	71.50 ± 1.13	71.03 ± 2.37^a^
	Boiled	65.20 ± 0.00	65.55 ± 0.49	65.05 ± 2.33	64.55 ± 0.21	65.18 ± 1.03^b^
	Fried	48.50 ± 0.14	54.65 ± 0.78	52.35 ± 0.21	53.20 ± 1.56	52.18 ± 2.52^e^
	Steamed	62.40 ± 0.00	65.35 ± 0.21	63.30 ± 0.99	61.45 ± 0.21	63.13 ± 1.59^c^
	Baked	61.10 ± 2.12	62.80 ± 0.57	61.10 ± 1.41	59.50 ± 1.56	61.13 ± 1.70^d^
	Grilled	63.15 ± 1.77	62.00 ± 0.71	58.35 ± 0.21	60.45 ± 2.19	60.99 ± 2.22^d^
	Average*	62.36 ± 7.82^b^	63.60 ± 5.17^a^	61.35 ± 5.31^b^	61.78 ± 5.85^b^	62.27 ± 5.99
Thigh	Fresh	74.22 ± 0.45	68.80 ± 0.99	67.25 ± 0.35	68.90 ± 3.11	69.79 ± 3.09^a^
	Boiled	70.39 ± 5.74	63.90 ± 0.85	64.05 ± 0.64	62.00 ± 0.85	65.09 ± 4.04^b^
	Fried	53.35 ± 0.64	57.15 ± 0.78	54.55 ± 1.91	48.45 ± 0.07	53.38 ± 3.47^d^
	Steamed	67.10 ± 1.13	64.10 ± 0.71	64.40 ± 1.13	62.25 ± 0.35	64.46 ± 1.97^b^
	Baked	61.85 ± 0.64	57.10 ± 0.14	61.90 ± 0.42	59.15 ± 0.92	60.00 ± 2.20^c^
	Grilled	63.90 ± 0.00	61.75 ± 2.33	58.65 ± 0.49	59.80 ± 0.28	61.03 ± 2.32^c^
	Average*	65.14 ± 7.17^a^	62.13 ± 4.39^b^	61.80 ± 4.41^b^	60.09 ± 6.44^c^	62.29 ± 5.84
Wing	Fresh	74.15 ± 0.21	69.15 ± 0.07	67.65 ± 1.06	64.70 ± 0.14	68.91 ± 3.68^a^
	Boiled	62.87 ± 0.30	62.15 ± 0.07	61.75 ± 0.21	60.15 ± 1.77	61.73 ± 1.27^b^
	Fried	48.92 ± 1.71	49.75 ± 0.49	48.30 ± 1.13	53.80 ± 3.35	50.19 ± 2.72^d^
	Steamed	62.75 ± 0.30	61.35 ± 0.07	62.14 ± 2.60	62.90 ± 1.13	62.29 ± 1.27^b^
	Baked	54.20 ± 0.00	56.00 ± 0.85	57.20 ± 0.85	54.95 ± 0.49	55.59 ± 1.30^c^
	Grilled	58.65 ± 0.07	56.20 ± 0.57	53.25 ± 0.49	54.90 ± 0.42	55.75 ± 2.14^c^
	Average*	60.26 ± 8.26^a^	59.10 ± 6.35^b^	58.38 ± 6.69^b^	58.57 ± 4.59^b^	59.08 ± 6.43
Total Average*		62.35 ± 7.58^a^	61.66 ± 5.41^b^	60.72 ± 5.36^c^	60.07 ± 5.50^d^	61.20 ± 6.05

* Numbers followed by the different superscript letter on the same line are significantly different in the ANOVA test (*p* > 0.05); ** Numbers followed by the different superscript letter on the same column are significantly different in the ANOVA test (*p* > 0.05).

### 3.2. The analysis of cholesterol

Table 2 shows cholesterol levels (mg/100 gm) in various chicken parts and processing methods across four cities. In general, the highest cholesterol levels were detected in grilled and fried chickens, particularly in the thighs and wings. Cholesterol levels in fresh chickens were generally lower than those in processed chickens. There was some variation in cholesterol levels across cities, but the differences between the processing methods were more significant. ANOVA results indicate significant differences across treatments (recognized by different superscripts).

**Table 2.** The average cholesterol levels of chicken meat from the four cities with different processing.

Part of Chicken Meat	Processing types	Cholesterol Content (mg/100 gm)				
		Medan	Bandung	Surabaya	Makasar	Average**
Whole	Fresh	123.44 ± 24.93	49.59 ± 7.38	45.75 ± 8.93	66.20 ± 19.54	71.24 ± 36.67^ab^
	Boiled	95.24 ± 7.74	87.92 ± 13.00	118.66 ± 14.72	142.06 ± 2.69	110.97 ± 24.71^ab^
	Fried	109.24 ± 39.09	101.29 ± 9.41	103.82 ± 34.06	134.03 ± 10.75	112.09 ± 28.29^ab^
	Steamed	95.13 ± 32.59	102.22 ± 54.76	87.23 ± 19.55	98.13 ± 49.33	95.68 ± 38.81^ab^
	Baked	155.43 ± 12.24	163.53 ± 48.72	121.59 ± 66.66	129.44 ± 4.53	142.50 ± 42.92^a^
	Grilled	83.44 ± 15.54	77.22 ± 2.57	102.24 ± 16.96	96.51 ± 67.08	89.85 ± 34.48^b^
	Average*	110.32 ± 33.31^a^	96.96 ± 45.56^a^	96.55 ± 39.99^a^	111.06 ± 42.20^a^	103.72 ± 39.83
Breast	Fresh	99.10 ± 3.23	75.68 ± 10.31	86.19 ± 20.41	47.84 ± 9.38	77.20 ± 23.04^ab^
	Boiled	99.10 ± 0.00	73.00 ± 5.04	108.75 ± 6.12	89.82 ± 8.90	92.66 ± 14.59^abc^
	Fried	87.77 ± 20.71	68.58 ± 6.15	121.64 ± 26.99	114.07 ± 20.56	98.01 ± 28.76^c^
	Steamed	104.51 ± 1.04	96.53 ± 12.46	107.42 ± 3.30	117.84 ± 8.59	106.57 ± 10.87^a^
	Baked	95.95 ± 13.80	83.74 ± 5.35	105.12 ± 7.76	80.201 ± 39.42	91.25 ± 22.34^bc^
	Grilled	127.03 ± 19.50	82.42 ± 1.55	116.42 ± 12.77	115.51 ± 6.66	110.34 ± 21.02^a^
	Average*	102.24 ± 17.06^a^	79.99 ± 11.58b	107.59 ± 17.59^a^	94.21 ± 31.34^ab^	96.01 ± 22.63
Thigh	Fresh	97.31 ± 4.61	132.37 ± 40.15	78.96 ± 4.15	64.05 ± 10.18	93.17 ± 33.52^a^
	Boiled	88.78 ± 35.14	90.54 ± 37.38	77.13 ± 24.29	150.08 ± 4.92	101.63 ± 40.16^a^
	Fried	124.83 ± 6.95	135.86 ± 24.73	113.33 ± 13.11	137.29 ± 63.34	127.83 ± 33.76^a^
	Steamed	113.27 ± 1.52	126.39 ± 12.03	134.91 ± 8.82	116.73 ± 26.25	122.82 ± 16.67^a^
	Baked	107.11 ± 3.02	122.44 ± 0.29	101.39 ± 7.44	116.26 ± 21.76	111.80 ± 13.80^a^
	Grilled	100.06 ± 31.51	112.81 ± 10.67	110.58 ± 1.62	150.86 ± 11.47	118.58 ± 26.03^a^
	Average*	105.22 ± 21.46^a^	120.07 ± 27.78^a^	102.72 ± 23.78^a^	122.54 ± 41.18^a^	112.64 ± 29.93
Wing	Fresh	78.15 ± 3.18	92.93 ± 1.44	87.07 ± 1.96	102.39 ± 11.68	90.13 ± 11.02^a^
	Boiled	73.47 ± 16.59	137.84 ± 14.15	102.21 ± 10.51	118.70 ± 13.89	108.05 ± 28.40^a^
	Fried	131.53 ± 9.50	152.08 ± 10.58	178.70 ± 38.44	172.56 ± 5.34	158.72 ± 27.39^a^
	Steamed	82.62 ± 27.79	118.55 ± 16.37	105.40 ± 7.98	111.38 ± 21.89	104.48 ± 22.93^ab^
	Baked	117.14 ± 12.09	169.45 ± 14.68	126.29 ± 5.94	140.00 ± 6.91	138.22 ± 23.29^a^
	Grilled	100.73 ± 11.81	99.73 ± 1.98	95.87 ± 5.83	121.15 ± 8.26	104.37 ± 12.79^b^
	Average*	97.27 ± 26.26^c^	128.43 ± 30.36^a^	115.92 ± 35.27^bc^	127.69 ± 26.65^ab^	117.33 ± 31.57
Total Average*		103.76 ± 24.90^a^	106.36 ± 35.86^a^	105.69 ± 30.35^a^	113.88 ± 37.13^a^	107.42 ± 32.40

* Numbers followed by the different superscript letter on the same line are significantly different in the ANOVA test (*p* > 0.05); ** Numbers followed by the different superscript letter on the same column are significantly different in the ANOVA test (*p* > 0.05).

## 4. Discussion

### 4.1. The water content

The present study demonstrated that the water content of chicken meat is strongly influenced by both the anatomical part and processing method, with significant differences observed across the four cities studied. Based on the chicken meat section, the water content of unprocessed (fresh) chicken breast meat was found to be the highest, followed by thigh meat. Consistent with previous research by Susanty et al. [31], which stated that every 100 gm of chicken meat contains a water content ranging from 68.73% to 73.80%, another study reported that the water content of fresh chicken meat ranges from 65.44% to 71.27% [19, 32, 33].

Another study reported that the muscle in chicken breast meat has a higher water content because it contains hydrophilic proteins (easily absorb water) and narrow, dense muscle fiber cavities (white meat), which readily absorb water [34].

After cooking, food ingredients have lower water content. The results of the analysis showed that all parts of the chicken meat that underwent processing (boiled, fried, steamed, grilled, and baked) experienced a decrease in water content compared to fresh chicken meat (unprocessed). Among all the processing methods, the largest decrease in water content was observed in fried chicken. This is because the frying process uses a high temperature of more than 175–190°C. The higher the cooking temperature, the greater the water loss from chicken meat [18, 19, 27, 32, 33]. A statistical study (ANOVA) confirmed that the differences were significant, especially between fresh and treated samples and among various processing procedures. The water content in the fried, steamed, grilled, and baked samples was not significantly different, likely because of the relatively elevated temperatures employed in these procedures, which facilitate considerable water loss.

### 4.2. The cholesterol content

Cholesterol is produced by the liver, but humans also obtain it from the food they consume. Although chicken is a low-fat food, it also contains cholesterol. The amount of cholesterol that enters the human body depends on the part of the chicken consumed and its processing. This study found that the cholesterol content of fresh chicken meat (unprocessed) varied significantly by sample origin; samples from Surabaya and Bandung differed from those from Medan and Makassar. Several factors, including handling of chickens before slaughter, genetics, breed, type of livestock, sex, age, feed, medications, supplements, hormones, antibiotics, minerals, and stress levels, influence cholesterol levels between cities [27, 34].

In the chicken meat section, the highest cholesterol content was found in the wings and the lowest in the breast. This is because chicken stores fat in different parts of the body; for example, chicken fat is mainly stored in the skin, and chicken thighs have higher fat and cholesterol contents than other parts of the meat. However, a statistical test showed that cholesterol levels across different parts of chicken meat were not significantly different. This was probably because the food provided was different. Feeds rich in fiber can reduce total cholesterol in chicken meat [35, 36].

Cooking processes that use heat can damage the nutritional content of food, such as denaturing proteins and reducing the water and fat content. Simple distinctions exist based on how they are processed. A statistical test revealed that cholesterol levels in boiled chicken were markedly different from those in fried, grilled, baked, and steamed chicken. The analysis showed that boiled and steamed chicken contained more moisture than fried, baked, or grilled chicken did. When water is boiled, it breaks down the fat. High temperatures also break down fat, so fat will come out and dissolve in boiling water [27].

Fried chicken had the highest cholesterol content among the processed meats, whereas wing meat had the highest cholesterol levels. Compared to fresh (unprocessed) chicken meat, cholesterol levels increased. This is suspected to be due to the absorption of cooking oil by chicken meat during frying [19, 27]. Another study showed that skinned wings contain more fat than other parts of the body (1.2% in the chest, 2.8% in the thighs, and 14.9% in the wings) [36]. The frying process causes more oil to be absorbed, increasing the saturated fat content and affecting the total cholesterol levels. Generally, cholesterol content increases after cooking, particularly after frying [37].

Chicken meat is a food ingredient expected to help maintain and reduce weight, as well as prevent cardiovascular disease, owing to its low cholesterol content. Replacing red meat with chicken can reduce the risk of cardiovascular disease by 19% [38]. The results of this study indicate that the processing method affects cholesterol levels in chicken meat. All methods of processing chicken meat (boiling, frying, steaming, grilling, and baking) increase cholesterol levels compared to fresh (unprocessed) chicken meat [39]. Therefore, boiled chicken is recommended because it causes the least increase in cholesterol levels.

## 5. Conclusions

The highest water content was observed in fresh chicken breast meat. In fresh samples, the thigh had the highest cholesterol content, while the whole part had the lowest. Among the processing methods, frying resulted in the greatest reduction in water content and the greatest increase in cholesterol levels. Boiling was the most effective method for minimizing cholesterol increase. Although some differences in cholesterol levels were observed between cities, the processing method had a more pronounced effect than the geographic origin.

## Data Availability

The data presented in this study are available from the corresponding author upon reasonable request.

## List of abbreviations

GC-FID/GC-MS, Gas chromatography-flame ionization detector; NPN, non-protein nitrogen; LDL, low-density lipoprotein; PUFAs, polyunsaturated fatty acids; TCMS, trimethylchlorosilane ether; DMF, dimethylformamide; ANOVA, Analysis of variance; dl, deciliter; gm, gram; h, hour; kg, kilogram; l, liter; mg, milligram; ml, milliliter; rpm, revolutions per minute.
